# Delay in pathological tissue processing time vs. mortality in oral cancer: Short communication

**DOI:** 10.1186/1758-3284-4-14

**Published:** 2012-04-26

**Authors:** Waseem Jerjes, Tahwinder Upile, Hani Radhi, Aviva Petrie, Aidan Adams, Jacqueline Callear, Panagiotis Kafas, Colin Hopper

**Affiliations:** 1 Department of Surgery, Dijla University College, Baghdad, Iraq; 2 Oral and Maxillofacial Surgery Unit, AL-Mustansirya University, Baghdad, Iraq; 3 UCL Department of Surgery, University College London, London, UK; 4 Leeds Institute of Molecular Medicine, University of Leeds, Leeds, UK; 5 Chase Farm & Barnet NHS Trust, Enfield, UK; 6 Head & Neck Unit, University College London Hospital, London, UK; 7 Biostatistics Unit, UCL Eastman Dental Institute, London, UK; 8 Department of Oral Surgery and Radiology, School of Dentistry, Aristotle University, Thessalonica, Greece

## Abstract

Several factors have been identified to affect morbidity and mortality in oral cancer patients. The time taken to process a resected cancer specimen in a patient presenting with primary or recurrent disease can be of interest as delay can affect earlier interventions post-surgery. We looked at this variable in a group of 168 consecutive oral cancer patients and assessed its relationship to mortality from the disease at 3 and 5 years. It is expected that delay in pathological processing time of surgical specimens acquired from patients with recurrent disease may increase or contribute to the increased rate of mortality. Further high evidence-based studies are required to confirm this.

## Introduction

The incidence of oral squamous cell carcinoma (OSCC) remains high. Oral and oro-pharyngeal carcinomas are the sixth most common cancer in the world. Numerous clinicopathological parameters have been implicated in prognosis, recurrence and survival, following this unforgiving disease [1,2].

The correct identification of pathology is essential to the correct treatment. Unfortunately due to the nature of replicative diseases, as in malignancy, tumour doubling time becomes an issue. Even small delays in applying further interventions may allow further tumour invasion and infiltration of the loco-regional surroundings resulting in a previously resectable growth becoming unresectable or unmanageable with the current chemo-radiotherapeutic protocols [1,2].

Pathological processing time is identified as the time taken from acquiring the resected tumour specimen by the surgeon to the reporting of the results by the histopathologist. The report will usually include grading and pathological staging of the tumour, the state of the surgical margins and any invasion to neurovascular or hard tissue structures.

It has been known that histopathological processing can take few days but this might increase if it involves composite specimen (i.e. hard tissue). Also the use of special testing can increase the time of processing. Over the last few years an increase in the workload on pathology departments has lead to further delay in pathological processing time [1].

The overall effects of delays in pathological processing time are unknown. In this short communication, we reviewed time intervals between taking the surgical specimen and definitive pathology report in patients with primary or recurrent disease. In each case we correlated this to 3- and 5-year survival rates.

## Materials and methods

This retrospective analytic study looked at 168 consecutive oral cancer patients who presented to University College London Hospital over a 10-year period (1992–2002). All patients suffered from recurrent disease. Proformas were created to collect the clinicopathological data and validated by a sample review.

Pathological processing time of the resected specimen was identified. “Duration 1” refers to the time (in days) taken to process and report on the resected tissue of the primary tumour. While “Duration 2” refers to the time (in days) taken to process and report on the resected tissue of the recurrent disease.

### Statistical analysis

The outcomes of the categorical clinicopathological variables were summarised as frequencies and percentages for the whole group of patients and for the recurrence group, categorised by 3 and 5 years survival and cause of death.

The duration of the pathological processing time of primary and recurrent disease was reported as means, standard deviations, minimal and maximal values. P-values at 3 and 5 years were generated when comparing pathological processing time in those who died from oral cancer and survived using an unpaired *t*-test.

## Results

The patient population comprised 113 males and 55 females; with 56.5 % Caucasians, 11.9 % Indians, 8.9 % Middle-Easterns, 6.5 % Africans and 7.7 % Caribbean backgrounds. Their mean age at the 1^st^ diagnosis of OSCC was 63.2 (SD4.6 years, Min 25 years, Max 94 years).

Primary sites were mainly identified in the tongue (50 %), floor of mouth (25 %), buccal mucosa (8 %) and alveolus (4 %). Tumour staging showed that half of the group had T1/T2 N0M0 disease while the other half had T3/T4 N0M0 disease. Treatment involved surgery alone in 12 patients, surgery and radiotherapy in 71 patients, surgery and radiochemotherapy in 26 patients, while the rest of the group received radiotherapy, chemotherapy, laser surgery or photodynamic therapy as the sole treatment at that time.

Overall survival at 3 years was 63.7 %. 71/113 males survived after 3 years from diagnosis compared to 36/55 females. Survival at 5-years (48.8 %) showed 53/113 alive males and 29/55 alive females. Cancer-related death showed 31 males died from loco-regional cancer spread and 15 who died from distant metastasis (i.e. pulmonary, hepatic), while 12 females died from loco-regional causes and 7 from distant cancer spread. Nearly all the patients who succumbed to the disease were from Caucasian and Indian backgrounds.

**Figure 1:**
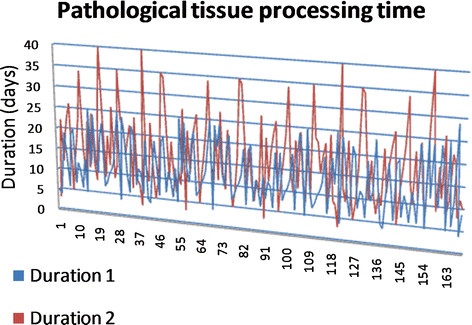
**Pathological processing time of the resected specimens.** “Duration 1” refers to the time (in days) taken to process and report on the resected tissue of the primary tumour. While “Duration 2” refers to the time (in days) taken to process and report on the resected tissue of the recurrent disease.

The mean pathological processing time of specimens acquired from primary-diseased patients (Duration 1) was reported as 13 days (SD6.5 days, Min 4 days, Max 29 days). The mean pathological processing time of specimens acquired from recurrent-diseased patients (Duration 2) was reported as 17 days (SD8.9 days, Min 3 days, Max 40 days). The 40-day duration has been seen in all patients who have had hard tissue resection. Figure 1 highlights the pathological processing time.

**Figure 2:**
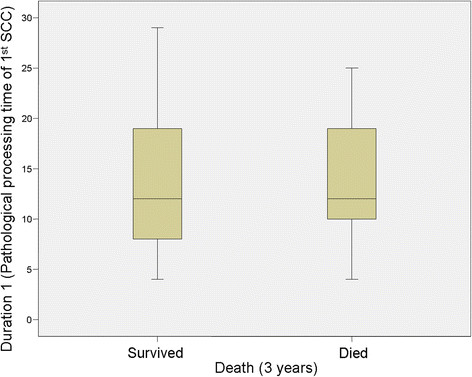
**Duration 1 versus survival at 3 years.** “Duration 1” refers to the time (in days) taken to process and report on the resected tissue of the primary tumour.

**Figure 3:**
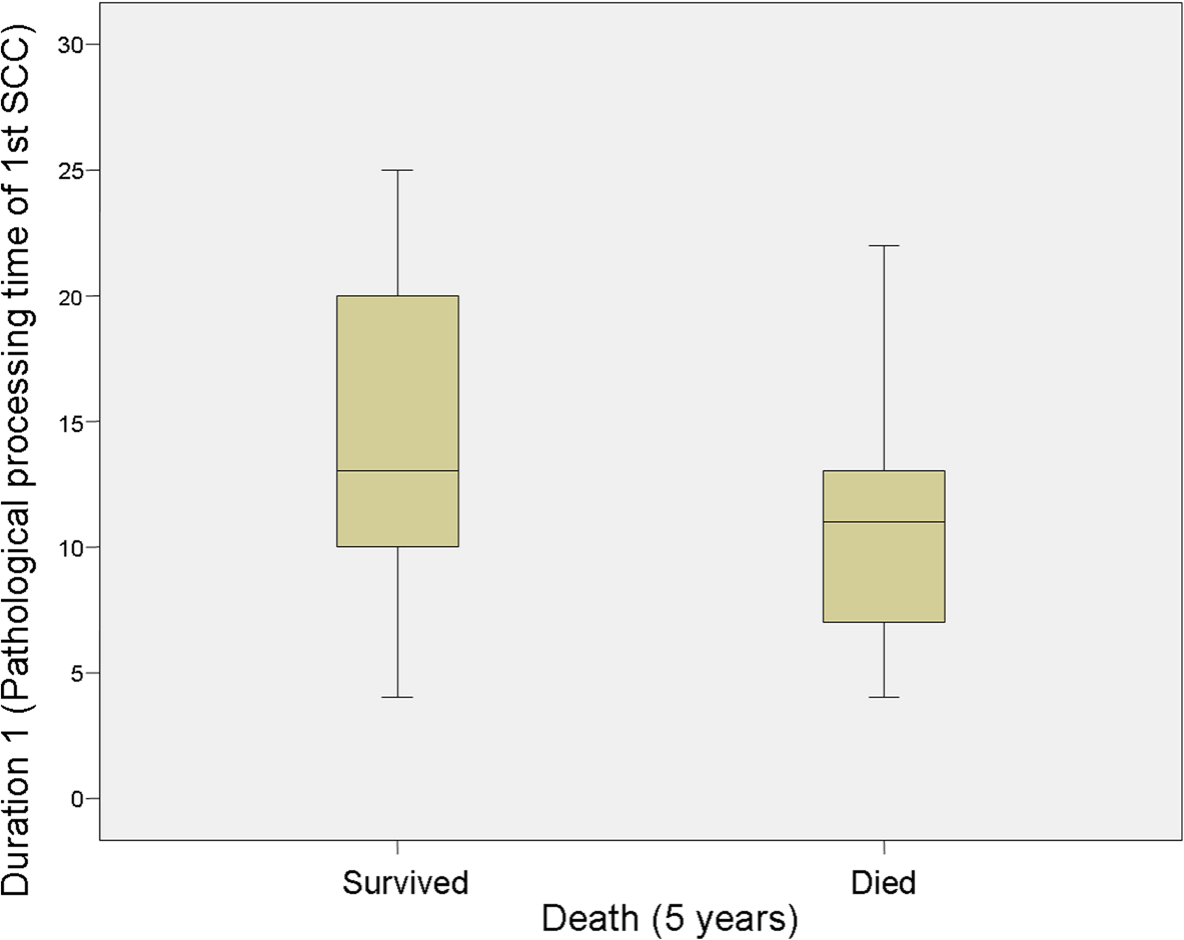
**Duration 1 versus survival at 5 years.** “Duration 1” refers to the time (in days) taken to process and report on the resected tissue of the primary tumour.

**Figure 4:**
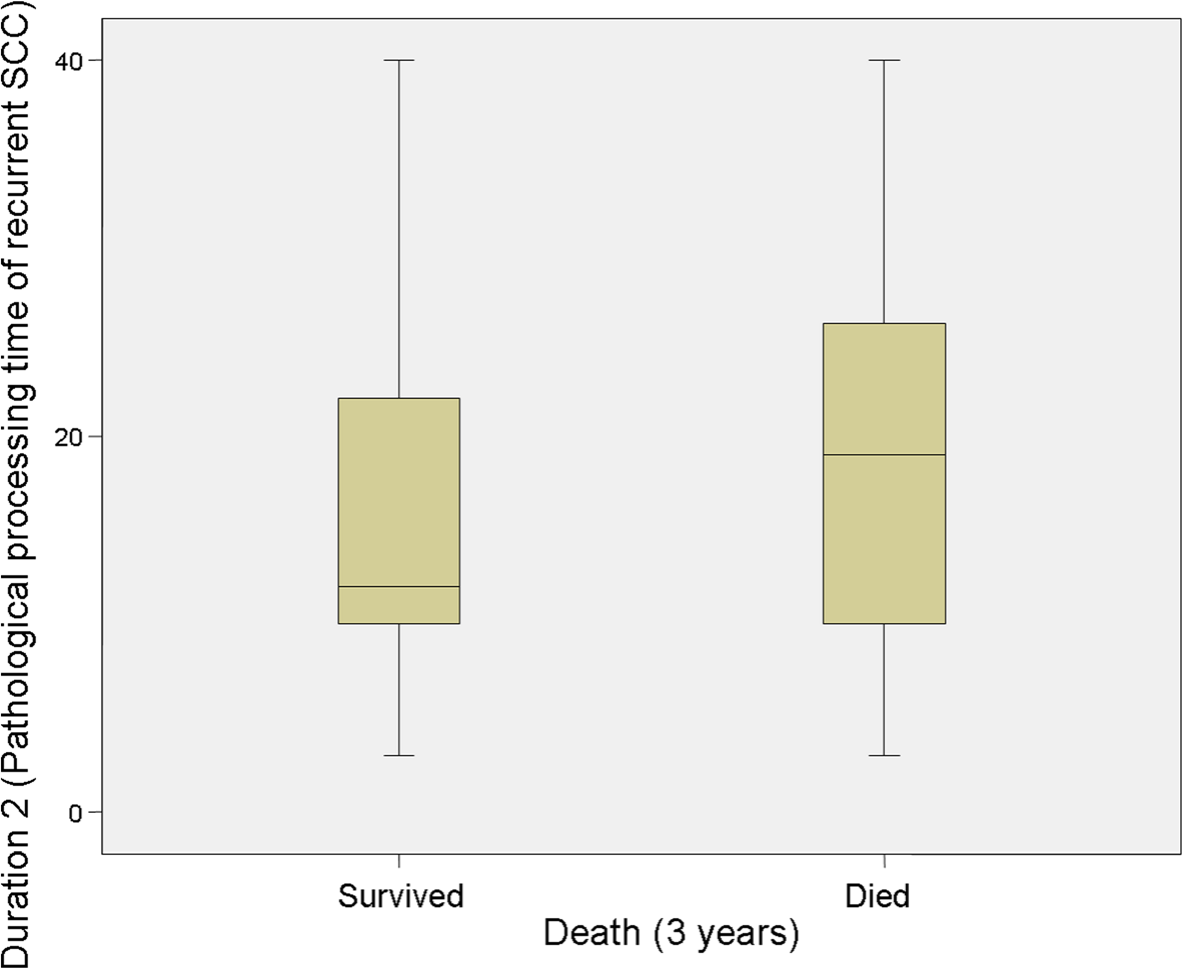
**Duration 2 versus survival at 3 years.** “Duration 2” refers to the time (in days) taken to process and report on the resected tissue of the recurrent disease.

**Figure 5:**
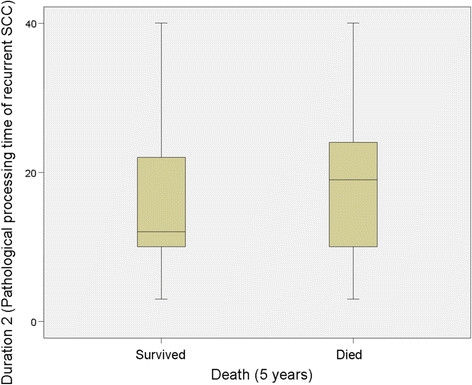
**Duration 2 versus survival at 5 years.** “Duration 2” refers to the time (in days) taken to process and report on the resected tissue of the recurrent disease.

No significant relationship was identified between Duration 1 (Pathological processing time of 1st SCC) and mortality at 3 years (*P* = 0.839) but there was a significant relationship at 5 years (*P* = 0.035), however this was clinically irrelevant (Table 1; Figures 2 and 3). For Duration 2 (pathological processing time of recurrent SCC), a significant relationship was identified at 3 years (*P* = 0.039) and 5 years (*P* = 0.033), (Table 1, Figure 4 and 5).

**Table 1:** Pathological processing time and mortality at 3 and 5 years

	Mean (days)	SD (days)	Min (Days)	Max (Days)	3 years survival P-value	5 years survival P-value
Duration 1	13	6.5	4	29	0.839	0.035
Duration 2	17	8.9	3	40	0.039	0.033

## Discussion

Squamous cell carcinoma of the oral cavity has a poor overall prognosis with a high tendency to recur at the primary site and extend to involve the cervical lymph nodes. Several clinicopathological parameters can be employed to assess outcome, recurrence and overall survival [1-3].

Despite evolution in management, the overall survival of patients has not improved significantly during the past 20 years, with 5-year survival rates between 45-50 %. Prognosis depends or varies with tumour primary site, nodal involvement, tumour thickness, and the status of the surgical margins. Moreover, the cumulative effects of tobacco, betel nut and alcohol decrease the survival rate [1-3].

As would intuitively be suggested, processing time is an important factor in cumulative prognosis but is especially significant in recurrent disease states. In recurrent disease, the host tumour interface is already compromised either as a consequence of the disease itself or its attendant treatment; hence tumours tend to be more aggressive in recurrence stage.

In patients with recurrent disease, previous interventions may have already breached the host barriers allowing tumour spread loco-regionally or not included suppressed tumour clones outside the original treatment field. In these cases, time is of the essence and we would commend meta-scheduling of the processing resected tissue in patients who have already had previous tumour treatment. However, it is worth highlighting the fact that resection specimens of recurrence disease tend to be more bulky as well as composite (i.e. include bone) increasing the possibility of processing time.

It is, also, likely that confounding factors as in immuno-suppression, tumour dedifferentiation, malnutrition, and treatment failure that would have lead to recurrence and hence a poor prognosis in this subgroup.

In conclusion, pathological processing time might be an important factor in oral cancer prognosis. It appears that delay in tissue processing time in patients with recurrent disease may contribute to the increased mortality rate. Further higher evidence-based studies are required to prove this and to identify other parameters that could influence morbidity and mortality when managing this unforgiving disease.

## Competing interests

The authors declare no competing interests.

## Authors' contributions

All authors have contributed intellectually and to the writing of this manuscript. AP: contributed to the statistical analysis of this study. All authors read and approved the final manuscript.
